# Salsolinol modulation of dopamine neurons

**DOI:** 10.3389/fnbeh.2013.00052

**Published:** 2013-05-24

**Authors:** Guiqin Xie, Krešimir Krnjević, Jiang-Hong Ye

**Affiliations:** ^1^Department of Anesthesiology, Pharmacology, and Physiology, New Jersey Medical School, University of Medicine and Dentistry of New JerseyNewark, NJ, USA; ^2^Department of Physiology, Nanjing Medical UniversityNanjing, China; ^3^Department of Physiology, McGill UniversityMontreal, QC, Canada

**Keywords:** reward, addictive property, electrophysiology, brain slices, mu opioid receptors, GABAergic transmission, glutamatergic transmissions, dopaminergic neurons

## Abstract

Salsolinol, a tetrahydroisoquinoline present in the human and rat brains, is the condensation product of dopamine and acetaldehyde, the first metabolite of ethanol. Previous evidence obtained *in vivo* links salsolinol with the mesolimbic dopaminergic (DA) system: salsolinol is self-administered into the posterior of the ventral tegmental area (pVTA) of rats; intra-VTA administration of salsolinol induces a strong conditional place preference and increases dopamine release in the nucleus accumbens (NAc). However, the underlying neuronal mechanisms are unclear. Here we present an overview of some of the recent research on this topic. Electrophysiological studies reveal that DA neurons in the pVTA are a target of salsolinol. In acute brain slices from rats, salsolinol increases the excitability and accelerates the ongoing firing of dopamine neurons in the pVTA. Intriguingly, this action of salsolinol involves multiple pre- and post-synaptic mechanisms, including: (1) depolarizing dopamine neurons; (2) by activating μ opioid receptors on the GABAergic inputs to dopamine neurons – which decreases GABAergic activity – dopamine neurons are disinhibited; and (3) enhancing presynaptic glutamatergic transmission onto dopamine neurons via activation of dopamine type 1 receptors, probably situated on the glutamatergic terminals. These novel mechanisms may contribute to the rewarding/reinforcing properties of salsolinol observed *in vivo*.

## Introduction

Alcohol/Ethanol is one of the most widely used drugs and the global burden of alcoholism is immense, with an estimated 3.8% (Rehm et al., 2009) or 3.2% (Spanagel et al., 2010) of worldwide deaths attributed to alcohol consumption. Accumulating evidence suggests that some addictive properties of alcohol are generated by its metabolites, such as acetaldehyde and its derivatives, notably salsolinol (SAL, 1-methyl-1,2,3,4-tetrahydro-6,7-dihydroxy-isoquinolines) (Deng and Deitrich, 2008). SAL is formed from dopamine: either by non-enzymatic Pictet-Spengler condensation with acetaldehyde, yielding racemic (R/S)-SAL, or by combination with pyruvic acid, followed by enzymatic decarboxylation and reduction, producing (R)-SAL. In the brains of mammals racemic (R/S)-SAL is formed by the Pictet-Spengler condensation (Rommelspacher et al., 1995; Haber et al., 1996). In the human brain, enantio-selective (R)-SAL can be synthesized from dopamine and acetaldehyde by (R)-SAL synthase (Naoi et al., 1998). (R/S)-SAL is present in biological fluids such as urine, plasma, cerebrospinal fluid and postmortem brains of both alcoholics and non-alcoholics (Sjöquist et al., 1982b; Haber et al., 1996). The effects of alcohol consumption on SAL concentrations in the biological fluids and the brain have been extensively reviewed by Hipolito et al. (2012).

Many studies have attempted to establish a correlation between alcohol ingestion and the increase of SAL levels in brain tissues. According to the majority, chronic alcohol exposure produces an increase in SAL levels in several brain regions such as the striatum, hypothalamus and limbic regions (Sjöquist et al., 1982a,b; Myers et al., 1985; Matsubara et al., 1987; Rojkovicova et al., 2008). The increase varied remarkably ranging from 0.08 (Starkey et al., 2006) to 7.59 pg/mg (Rojkovicova et al., 2008). With one exception (Haber et al., 1996), previous human studies found that, after acute (Faraj et al., 1989; Rommelspacher et al., 1995) or chronic (Faraj et al., 1989) alcohol drinking, SAL levels rise in plasma (and presumably brain). Recent evidence that SAL in the brain accounts for some aspects of alcohol's addictive properties have been elegantly reviewed (Hipolito et al., 2012; Deehan et al., 2013a,b); but still little is known about how SAL acts at the cellular level.

The midbrain ventral tegmental area (VTA) has been extensively studied as a target for the central effects of alcohol (Morikawa and Morrisett, 2010), its first metabolite acetaldehyde (Melis et al., 2009; Karahanian et al., 2011), as well as its metabolic derivative SAL (Hipolito et al., 2012). The majority of neurons in the VTA are either dopaminergic (DA) or GABAergic (Lacey et al., 1989; Yung et al., 1991; Johnson and North, 1992b; Chieng et al., 2011), with only a few glutamatergic neurons (Nair-Roberts et al., 2008). The VTA DA neurons project mainly to the nucleus accumbens (NAc) and prefrontal cortex (PFC) (Oades and Halliday, 1987). Dopamine is involved in self-administration of most drugs of abuse, and drugs abused by humans increase dopamine output in target regions of the brain (Di Chiara and Imperato, 1988; Volkow et al., 2007). Addictive substances of different types modulate DA neuron activity and dopamine release from the VTA (Lüscher and Malenka, 2011). Recently, we employed patch clamp in combination with pharmacological techniques to investigate SAL's immediate effects on VTA DA neurons in rat brain slices (Xie and Ye, 2012; Xie et al., 2012). We found that SAL (0.01–1 μM) significantly stimulates the activity of DA neurons. In this review, we will focus on the multiple underlying cellular mechanisms, in order to clarify how SAL modulates neuronal excitability in the VTA.

## SAL's psychoactive effects in the mesolimbic dopamine system *In vivo*

Research from several laboratories has led to the idea that SAL participates in ethanol's psychoactive effects in rodents through its own rewarding properties. Early animal studies revealed that SAL promotes alcohol drinking (Duncan and Deitrich, 1980; Myers et al., 1982). Recent data from several groups support the notion that SAL is responsible for some addiction-related psychoactive behaviors related to the mesolimbic dopamine system. Indeed, in Wistar rats, microinjections of SAL (5, 25 μM) into the NAc core increase local dopamine extracellular levels (measured by HPLC), whereas the same doses of SAL injected into the NAc shell significantly reduced the dopamine levels in that subregion (Hipolito et al., 2009). However, microinjection of SAL into the posterior VTA increased DA levels in the ipsilateral accumbens shell by 41% (Hipolito et al., 2009). Recently, Deehan et al. (2013a,b) also reported that SAL stimulates dopamine release in the posterior ventral tegmental area (pVTA). In this study, the effects of SAL on dopamine release were dose-dependent, in an inverted U-shape manner, with 0.3 μM SAL producing a peak dopamine efflux (to 300% of baseline) and higher concentrations (3 μM) a significantly lower response (Deehan et al., 2013a,b). In parallel with these neurochemical findings, SAL elicited some behavioral effects. Direct injection of only 30 pmol SAL into the pVTA resulted in behavioral sensitization and induced strong motor activity in rats (Hipolito et al., 2010). Moreover, significant place preference was induced by SAL, given either intraperitoneally (ip) (Matsuzawa et al., 2000) or by local microinjection into the pVTA (Hipolito et al., 2011). Rodd and colleagues found that rats readily self-administered SAL into the NAc shell (Rodd et al., 2003, 2008) and pVTA (Rodd et al., 2008). Such reinforcing actions seem to depend on activation of DA neurons, being reduced by co-infusion of quinpirole [a D (2, 3) receptor agonist] (Rodd et al., 2008). Below, we will discuss the possible cellular mechanisms underlying these psychoactive effects of SAL, in the light of our recent findings in brain slices *in vitro.*

### SAL depolarizes pVTA DA neurons *In vitro* and accelerates their discharge

Under current-clamp, SAL was found to depolarize the membrane potential of VTA DA neurons in rats (Xie et al., 2012). In keeping with this depolarization, SAL increased the firing rate of DA neurons in a reversible and dose-dependent manner, with a peak effect at 0.1 μM. This dose dependence, however, was biphasic: at concentrations of 0.01–0.1 μM, the firing rate increased with SAL concentration; but at higher concentrations, the increase diminished sharply. Such concentrations are within a pharmacologically relevant range (Matsubara et al., 1987; Haber et al., 1999). This inverted, U-shaped concentration response curve seen *in vitro* is remarkably similar to the concentration dependence of SAL intra-pVTA microinjections effect on locomotor activity: this dose-response curve had an inverted U-shaped profile, with a peak at 30 pmol (Hipolito et al., 2010). Rodd et al. found that 0.03–0.3 μM SAL was readily self-administered when injected directly into the posterior, not anterior VTA of Wistar rats (Rodd et al., 2008). Notably, our *in vitro* and those *in vivo* experiments revealed that SAL is far more potent than ethanol as stimulator of DA neurons in the pVTA. In contrast to the strong activation induced by 0.1 μM SAL, ethanol had a similar effect only at concentrations of 100–200 mM (Brodie et al., 1990; Xiao et al., 2007). Thus, SAL is 1–2 million times more effective than ethanol as stimulator of DA neurons in the pVTA.

## GABAergic and glutamatergic transmissions in the VTA play a critical role in SAL's effect

### Disinhibition through the activation of μ opioid receptors (MORs) on GABAergic afferents

The projection neurons in the VTA, mostly DA neurons, are under inhibitory GABAergic control. Several GABAergic inputs are known, including those from GABA neurons in the rostomedial tegmental nucleus (RMTg) (Barrot et al., 2012), local GABA neurons (i.e., interneurons) (Johnson and North, 1992a; Lüscher and Malenka, 2011; Omelchenko et al., 2009; Tan et al., 2012), medium spiny neurons of the NAc and the ventral pallidum (Kalivas, 1993; Kalivas et al., 1993; Hjelmstad et al., 2013). Both GABA_A_ and GABA_B_ receptors mediate the inhibitory action of GABA on DA neurons (Johnson and North, 1992a; Brazhnik et al., 2008; Theile et al., 2011). Both *in vivo* and *in vitro*, blockade of GABA_A_Rs strongly increases DA cell firing (Johnson and North, 1992a; Xiao et al., 2007; Matsui and Williams, 2011; Theile et al., 2011; Guan et al., 2012); GABAergic IPSCs therefore normally dampen the excitability of DA neurons. GABA_A_R blockade in the VTA increases dopamine levels in the NAc (Ikemoto et al., 1997) and is strongly rewarding (Laviolette and van der Kooy, 2001). Several lines of evidence have linked ethanol-induced reinforcement to the GABAergic system in the VTA. For example, VTA GABA neurons become hyperexcitable during ethanol withdrawal (Gallegos et al., 1999). Both systemic and intra-VTA administration of GABA_A_R agonists facilitate, whereas antagonists decrease, voluntary ethanol drinking in rats (Smith et al., 1992). In line with these *in vivo* studies, we have recently reported several relevant findings obtained *in vitro*, during recordings of neuronal activity in brain slices from rats (Xiao et al., 2007; Xiao and Ye, 2008). Thus, ethanol inhibited GABA neurons (through activation of MORs); it enhanced DA neuron firing. Moreover, GABA_A_ antagonists such as bicuculline and gabazine, attenuate the ethanol-induced increase in firing of VTA-DA neurons. Further tests revealed an involvement of μ opioid receptors (MORs) as the MOR agonist DAMGO and MOR antagonist naltrexone significantly attenuated the increase in firing induced by ethanol and even altered the basal firing rate of the DA neurons, indicating ongoing opioid modulation (Xiao et al., 2007; Xiao and Ye, 2008). By contrast, Theile (Theile et al., 2011) reported that while ethanol-acceleration of the firing rate of VTA DA neurons was increased by picrotoxin, an antagonist of GABA_A_ and glycine receptors, it was unaffected by naltrexone, and DAMGO did not change the ongoing firing. This apparent difference suggests some links between MORs, GABA_A_Rs and the effect of ethanol on DA neurons.

Compared to the role of GABA in ethanol abuse, we know much less about how SAL affects GABAergic transmission. Previous studies have found that SAL is a morphine-like alkaloid. It binds to opioid receptors and has opioid-like effects (Fertel et al., 1980; Lucchi et al., 1982). We found (Xie et al., 2012) that both gabazine and naltrexone reduce the acceleration of DA neuronal firing produced by SAL, suggesting that SAL's action may be mediated via MOR on GABAergic neurons. In support of this idea, SAL reduced the frequency of spontaneous IPSCs recorded in DA neurons, without changing their amplitude; but SAL decreased the size of evoked IPSCs and increased the paired-pulse ratio. These observations indicate that SAL depresses GABAergic transmission to DA neurons by an opioid sensitive presynaptic mechanism. Indeed, SAL's effect on sIPSCs was suppressed by naltrexone or DAMGO. MORs are enriched in the VTA and are primarily located on non-DA neurons (Mansour et al., 1995), the RMTg and its efferents to the DA neurons (Jhou et al., 2009; Jalabert et al., 2011; Matsui and Williams, 2011; Hjelmstad et al., 2013). It is generally believed that MORs–mediated inhibition of GABAergic neurons leads to excitation of DA neurons by a disinhibitory mechanism (Johnson and North, 1992a; Jalabert et al., 2011; Matsui and Williams, 2011). In keeping with this idea, both systemic and intra-VTA administrations of MOR agonists increase VTA DA neuron firing and NAc dopamine release (Matthews and German, 1984; Latimer et al., 1987; Di Chiara and Imperato, 1988; Leone et al., 1991; Spanagel et al., 1992). Previous experiments *in vivo* have shown that SAL-associated place preference was blocked by intraperitoneal or local administration of β-Funaltrexamine, an antagonist of MORs (Matsuzawa et al., 2000). Local pretreatment with β-Funaltrexamine hydrochloride also prevented the SAL-evoked increase in NAc dopamine levels (Hipolito et al., 2011). Our observation of pronounced effects of naltrexone on GABAergic IPSCs is strong evidence of ongoing opioid release in the VTA (Xiao et al., 2007; Xiao and Ye, 2008; Xie et al., 2012). Moreover, naltrexone largely eliminated the effects of SAL on DA neurons. The simplest explanation of our results is that SAL activates MORs on GABAergic neurons or their efferents, thus exciting VTA DA neurons by disinhibition. Overall, our *in vitro* findings are consistent with the notion that SAL's excitatory effects in rat pVTA are mediated, at least partly, by activation of MORs (Hipolito et al., 2011) and the resulting suppression of GABAergic inhibition. How SAL activates MORs remains to be clarified. Though known to bind to opioid receptors, it has only a low affinity, significant binding requiring relatively high micromolar concentrations of SAL (Fertel et al., 1980; Lucchi et al., 1982). The binding of SAL lowers the binding of endogenous opioids. Therefore, other mechanisms of SAL action at very low concentrations, such as enhanced release or slower removal of endogenous opioid, should also be considered.

### Glutamatergic transmission to VTA is enhanced by sal-induced activation of D1Rs

VTA DA neurons receive numerous glutamatergic afferents from many parts of the brain, including the PFC (Sesack et al., 2003; Geisler et al., 2007) and subcortical structures, such as the pedunculopontine (PPTg) and laterodorsal tegmental (LDTg) nuclei (Charara et al., 1996; Clements et al., 1991; Lavoie and Parent, 1994), the bed nucleus of the stria terminals (BNST) (Georges and Aston-Jones, 2002), the superior colliculus (SC) (Comoli et al., 2003; Dommett et al., 2005), the lateral hypothalamic and preoptic areas, periaqueductal gray, the dorsal and median raphe (Geisler et al., 2007), as well as the lateral habenula (Gonçalves et al., 2012). Some glutamatergic neurons are also present within the VTA (Dobi et al., 2010). Excitatory synaptic inputs which activate α-amino-3-hydroxy-5-methyl-4-isoxazolepropionic acid (AMPA), and NMDA-type ionotropic glutamate receptors in DA neurons are a key component in the regulation of DA cell excitability (Overton and Clark, 1997). Accordingly, iontophoretic applications of both AMPA and NMDA receptor agonists can stimulate DA neuron firing (Christoffersen and Meltzer, 1995; Zhang et al., 1997). Consistently, applications of APV + DNQX (AMPA and NMDA receptor antagonists, respectively) slightly but significantly lower the firing rate of DA neurons (Xie and Ye, 2012).

Glutamatergic transmission plays an important role in the effects of ethanol (Eckardt et al., 1998; Krystal et al., 2003). In several brain regions, ethanol inhibits NMDA and non-NMDA glutamate receptors, as well as glutamate release (Siggins et al., 2005). However, ethanol can increase glutamate release under some circumstances. Systemic administration of ethanol increases glutamate release in the NAc of low-alcohol sensitive rats (Dahchour et al., 2000) and addiction-prone Lewis rats (Selim and Bradberry, 1996). Acute ethanol administration increases glutamate release in the central nucleus of the amygdala from rats receiving chronic ethanol treatment (Roberto et al., 2004; Zhu et al., 2007). Both acute and repeated exposure to low doses of ethanol raised glutamate levels in the pVTA (Ding et al., 2012). We have previously reported that ethanol enhances glutamatergic transmission to VTA DA neurons (Deng et al., 2009; Xiao et al., 2009). Most recently, we found that SAL also enhances glutamatergic transmission to DA neurons in the VTA, increasing the frequency of both firing and spontaneous EPSCs (Xie and Ye, 2012). The application of APV + DNQX substantially attenuated SAL's action on firing, indicating a very substantial glutamatergic component in SAL-induced excitation of DA neurons. The increase in frequency of spontaneous EPSCs was abolished by the sodium channel blocker tetrodotoxin, indicating that SAL's effect involved voltage-dependent Na^+^ channels. Since SAL did not alter the amplitude of either sEPSCs or mEPSCs, but increased the EPSC_2_/EPSC_1_ ratio during paired-pulse stimulation, its site of action was probably presynaptic. These findings clearly point to the involvement of glutamatergic transmission in SAL's effects in VTA. Dopamine receptors consist of D1-like (D1 and D5 receptors) and D2-like (D2, D3, and D4 receptors) families. Both D1R and the D2R family (D2R in particular) have been implicated in the mechanisms of drug dependence and abuse (Blum et al., 1990). Disruption of D1R gene expression (El-Ghundi et al., 1998) or administration of a D1R antagonist (Liu and Weiss, 2002) attenuates or prevents alcohol-seeking behavior. In the VTA, D1Rs are expressed on glutamatergic axons (Lu et al., 1997) but not on the soma of VTA DA neurons (Mansour et al., 1992; Lu et al., 1997). The activation of D1Rs increases glutamate levels in the VTA (Kalivas and Duffy, 1995). In our experiments, SKF83566 (a selective D1R antagonist), suppressed SAL's action on evoked EPSCs in VTA, confirming that the enhancement of glutamatergic transmission was mediated by D1Rs. We also found that SKF83566 attenuated the SAL-induced acceleration of DA neuron firing. SAL may thus increase the somatodendritic release of dopamine; and by raising the extracellular level of dopamine may retrogradely activate the D1Rs on the glutamate-releasing terminals, which in turn increases glutamate release and the excitability of DA neurons. Somatodendritic release of dopamine in the midbrain DA neurons is Na^+^ channel-dependent (Threlfell and Cragg, 2007). In keeping with this, we found that the increase in the frequency of spontaneous EPSCs by SAL was abolished by tetrodotoxin. SAL's D1R-dependent effect on glutamatergic transmission is similar to that we observed previously with ethanol (Deng et al., 2009; Xiao et al., 2009); but SAL is much more potent than ethanol (the effective concentrations being 0.1 μM for SAL vs. 40 mM for ethanol). This is consistent with both *in vivo* and *in vitro* findings that the DA system is particularly sensitive to SAL (Hipolito et al., 2011; Rodd et al., 2008; Xie and Ye, 2012).

We showed that the ethanol-induced increase in the release of glutamate (EPSCs) in the VTA was eliminated when dopamine was depleted by pretreatment with reserpine (Deng et al., 2009; Xiao et al., 2009). Since SAL is the condensation product of dopamine and acetaldehyde, depletion of dopamine could prevent the formation of SAL. This could explain why ethanol failed to increase glutamatergic transmission when dopamine was depleted.

## Summary

**Figure 1:**
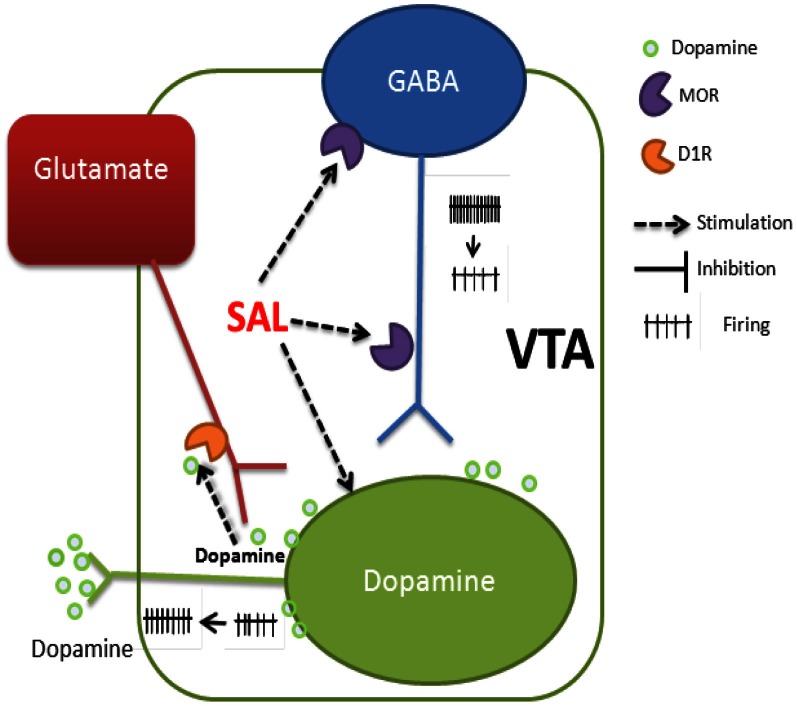
**Schematic representation of the mechanisms underlying SAL's excitation of putative DA neurons in rat Pvta.** (1) Depolarizing the membrane and increasing the firing rate. (2) Activation of MORs on the GABAergic neurons and/or their afferents reduces GABA release onto DA neurons. (3) Activation of D1Rs at the glutamatergic afferents increases glutamate release onto DA neurons. VTA, ventral tegmental area.

Our *in vitro* findings in combination with *in vivo* experiments by other groups—reviewed here—identify cellular mechanisms underlying SAL's psychoactive effects on the mesolimbic dopamine system. SAL's stimulating action in the pVTA involves modulation of synaptic inputs and intrinsic properties of DA neurons (schematically depicted in Figure 1): (1) depolarizing DA neurons and increasing their firing rate; (2) activating MORs on the GABAergic neurons, which inhibit GABAergic transmission to VTA DA neurons (resulting in disinhibition); (3) enhancing glutamatergic transmission to DA neurons by activating D1Rs situated on glutamatergic afferents. Thus, by acting on both presynaptic and postsynaptic targets on the DA neurons, SAL enhances the discharge of VTA DA neurons and so increases dopamine release in the downstream brain regions. Whatever its precise role—whether as detector of rewarding stimuli (Mirenowicz and Schultz, 1996) or modulating network activity in PFC (Durstewitz et al., 2000; González-Burgos et al., 2002; Lapish et al., 2007)—it is clear that the mesocortical DA system is important in processes leading to addiction. Hence, the novel mechanisms proposed here may contribute to the rewarding properties of SAL observed *in vivo*. Understanding how SAL affects the activity of VTA dopamine neurons could have profound implications for the prevention and treatment of alcoholism.

### Conflict of interest statement

The authors declare that the research was conducted in the absence of any commercial or financial relationships that could be construed as a potential conflict of interest.
